# Supplementary material for: Poly(dA:dT)-Rich DNAs Are Highly Flexible in the Context of DNA Looping
Source: PLoS One. 2013 Oct 11;8(10):e75799. doi: 10.1371/journal.pone.0075799 (PMC3795714; doi:10.1371/journal.pone.0075799)
Supplement: File S1 — Supporting figures. Figure S1 “No-promoter” looping sequences used in this work. Figure S2 “With-promoter” looping sequences used in this work. Figure S3 Sequence-dependent twist stiffness. Figure S4 Looping probabilities and J-factors for the two looped states separately. Figure S5 Tether lengths of looped and unlooped states as a function of loop length and sequence. Figure S6 Tether length as a function of J-factor. (PDF) [file pone.0075799.s001.pdf]

# Poly(dA:dT)-rich DNAs are highly flexible in the context of DNA looping: Supporting Information

Stephanie Johnson*, Yi-Ju Chen*, and Rob Phillips

## List of Figures

S1 "No-promoter" looping sequences used in this work. . . . . . 2
S2 "With-promoter" looping sequences used in this work. . . . . . 3
S3 Sequence-dependent twist stiffness. . . . . . 4
S4 Looping probabilities and J-factors for the two looped states separately. . . . . . 5
S5 Tether lengths of looped and unlooped states as a function of loop length and sequence. . . 6
S6 Tether length as a function of J-factor. . . . . . 7

*These authors contributed equally to this work.

```
E8108: GGCCGGGCTGCTGCGTAGAACTACTTTTATTTATCGCCTCCACGGTGCTGATCCCCTGTGCTGTTGGCCGTGTTATCTCGAGTTAGTACGACGTCCGCCAGCCGACGC

TA108: GGCCGTTAATTGGTCGTAGCAAGCTCTAGCACCGCTTAAACGCACGTACGCGCTGTCTACCGCGTTTTAACCGCCAATAGGATTACTTACTAGTCTCTAGGCACGTGC

5S101: -------CATAACATCCCTGACCCTTTAAATAGCTTAACTTTCATCAAGCAAGAGCCTACGACCATACCATGCTGAATATACCGGTTCTCGTCCGATCACCGAAGTCA
5S102: ------TCATAACATCCCTGACCCTTTAAATAGCTTAACTTTCATCAAGCAAGAGCCTACGACCATACCATGCTGAATATACCGGTTCTCGTCCGATCACCGAAGTCA
5S103: -----GTCATAACATCCCTGACCCTTTAAATAGCTTAACTTTCATCAAGCAAGAGCCTACGACCATACCATGCTGAATATACCGGTTCTCGTCCGATCACCGAAGTCA
5S104: ----CGTCATAACATCCCTGACCCTTTAAATAGCTTAACTTTCATCAAGCAAGAGCCTACGACCATACCATGCTGAATATACCGGTTCTCGTCCGATCACCGAAGTCA
5S105: ---ACGTCATAACATCCCTGACCCTTTAAATAGCTTAACTTTCATCAAGCAAGAGCCTACGACCATACCATGCTGAATATACCGGTTCTCGTCCGATCACCGAAGTCA
5S106: --GACGTCATAACATCCCTGACCCTTTAAATAGCTTAACTTTCATCAAGCAAGAGCCTACGACCATACCATGCTGAATATACCGGTTCTCGTCCGATCACCGAAGTCA
5S107: -TGACGTCATAACATCCCTGACCCTTTAAATAGCTTAACTTTCATCAAGCAAGAGCCTACGACCATACCATGCTGAATATACCGGTTCTCGTCCGATCACCGAAGTCA
5S108: ATGACGTCATAACATCCCTGACCCTTTAAATAGCTTAACTTTCATCAAGCAAGAGCCTACGACCATACCATGCTGAATATACCGGTTCTCGTCCGATCACCGAAGTCA

PolyA101:  ACCTTGTATTGTATTTCCTTTGCGTGATGAAAAAAAAACTGAAAAAGAGAAAAATAAGAAAATCTTCTAGAACGTTCCGAAACAGGAC-gtgctgatcccct------
PolyA102:  ACCTTGTATTGTATTTCCTTTGCGTGATGAAAAAAAAACTGAAAAAGAGAAAAATAAGAAAATCTTCTAGAACGTTCCGAAACAGGAC-gtgctgatcccctg-----
PolyA103:  ACCTTGTATTGTATTTCCTTTGCGTGATGAAAAAAAAACTGAAAAAGAGAAAAATAAGAAAATCTTCTAGAACGTTCCGAAACAGGAC-gtgctgatcccctgt----
PolyA104:  ACCTTGTATTGTATTTCCTTTGCGTGATGAAAAAAAAACTGAAAAAGAGAAAAATAAGAAAATCTTCTAGAACGTTCCGAAACAGGAC-gtgctgatcccctgtg---
PolyA105:  ACCTTGTATTGTATTTCCTTTGCGTGATGAAAAAAAAACTGAAAAAGAGAAAAATAAGAAAATCTTCTAGAACGTTCCGAAACAGGAC-gtgctgatcccctgtgc--
PolyA106:  ACCTTGTATTGTATTTCCTTTGCGTGATGAAAAAAAAACTGAAAAAGAGAAAAATAAGAAAATCTTCTAGAACGTTCCGAAACAGGAC-gtgctgatcccctgtgct-
PolyA107:  ACCTTGTATTGTATTTCCTTTGCGTGATGAAAAAAAAACTGAAAAAGAGAAAAATAAGAAAATCTTCTAGAACGTTCCGAAACAGGACggtgctgatcccctgtgct-
PolyA108:  ACCTTGTATTGTATTTCCTTTGCGTGATGAAAAAAAAACTGAAAAAGAGAAAAATAAGAAAATCTTCTAGAACGTTCCGAAACAGGACggtgctgatcccctgtgctg

CG101: -------GGGGCACCCACCAGATGCCAGCTGGAGCTCTCCTGTATGAGGGATCTGTTGATTCCAGCTGGGAGGTGTCTGCTACTCAGGAGGCACAGTTGTCAGGGACC
CG102: ------AGGGGCACCCACCAGATGCCAGCTGGAGCTCTCCTGTATGAGGGATCTGTTGATTCCAGCTGGGAGGTGTCTGCTACTCAGGAGGCACAGTTGTCAGGGACC
CG103: -----GAGGGGCACCCACCAGATGCCAGCTGGAGCTCTCCTGTATGAGGGATCTGTTGATTCCAGCTGGGAGGTGTCTGCTACTCAGGAGGCACAGTTGTCAGGGACC
CG104: ----AGAGGGGCACCCACCAGATGCCAGCTGGAGCTCTCCTGTATGAGGGATCTGTTGATTCCAGCTGGGAGGTGTCTGCTACTCAGGAGGCACAGTTGTCAGGGACC
CG105: ---CAGAGGGGCACCCACCAGATGCCAGCTGGAGCTCTCCTGTATGAGGGATCTGTTGATTCCAGCTGGGAGGTGTCTGCTACTCAGGAGGCACAGTTGTCAGGGACC
CG106: --CCAGAGGGGCACCCACCAGATGCCAGCTGGAGCTCTCCTGTATGAGGGATCTGTTGATTCCAGCTGGGAGGTGTCTGCTACTCAGGAGGCACAGTTGTCAGGGACC
CG107: -TCCAGAGGGGCACCCACCAGATGCCAGCTGGAGCTCTCCTGTATGAGGGATCTGTTGATTCCAGCTGGGAGGTGTCTGCTACTCAGGAGGCACAGTTGTCAGGGACC
CG108: TTCCAGAGGGGCACCCACCAGATGCCAGCTGGAGCTCTCCTGTATGAGGGATCTGTTGATTCCAGCTGGGAGGTGTCTGCTACTCAGGAGGCACAGTTGTCAGGGACC
```

Figure S1: "No-promoter" looping sequences used in this work, compared to one length each of the E8 and TA sequences used in [1] (see Ref. [1] for additional lengths of the E8 and TA sequences). All sequences are listed 5′ to 3′. The $O_{id}$ operator is immediately 5′ to these sequences, and has the sequence 5′-AATTGTGAGCGCTCACAATT-3′. $O_1$ is immediately 3′ and has the sequence 5′-AATTGTGAGCGGATAACAATT-3′. The 5S sequences shown here are the middle 101-108 bp of the full 5S sequence described by [2]; the CG sequences are the middle 101-108 bp from the Y-chromosome of "Human 2" at http://genie.weizmann.ac.il/pubs/field08/field08_data.html (see also Ref. [3]). The poly(dA:dT)-rich sequence from [4] is only 88 bp long and so was padded with E8 on the $O_1$-proximal end (as indicated by the lower-case letters). Poly(dA:dT) tracts, defined as stretches of 4 or more A bases in a row, are indicated in green; the TA/AA/AT/TT bases spaced ten bases apart that contribute to the nucleosome preferences of the TA and 5S sequences are indicated in red; and the CG/GG/CC/GC bases five bases out of phase with the TA/AA/AT/TT bases, which also contribute to nucleosome preference, are shown in blue. Note that the TA and GC bases on the 3′ end of the TA sequence, in boldface letters, are one base-pair out of phase with those on the 5′ end.

```
E8108(prom): --------TACTTTTATTTATCGCCTCCACGGTGCTGATCCCCTGTGCTGTTGGCCGTGTTATCTCGAGTTAGTACGACC---------------

TA108(prom): ----------CTCTAGCACCGCTTAAACGCACGTACGCGCTGTCTACCGCGTTTTAACCGCCAATAGGATTACTTACTAGTC-------------

5S101(prom): -----------------------------------TAACTTTCATCAAGCAAGAGCCTACGACCATACCATGCTGAATATACCGGTTCTCGTCCGATCAC--------
5S102(prom): ----------------------------------TTAACTTTCATCAAGCAAGAGCCTACGACCATACCATGCTGAATATACCGGTTCTCGTCCGATCAC--------
5S103(prom): ---------------------------------CTTAACTTTCATCAAGCAAGAGCCTACGACCATACCATGCTGAATATACCGGTTCTCGTCCGATCAC--------
5S104(prom): --------------------------------GCTTAACTTTCATCAAGCAAGAGCCTACGACCATACCATGCTGAATATACCGGTTCTCGTCCGATCAC--------
5S105(prom): -------------------------------AGCTTAACTTTCATCAAGCAAGAGCCTACGACCATACCATGCTGAATATACCGGTTCTCGTCCGATCAC--------
5S106(prom): ------------------------------TAGCTTAACTTTCATCAAGCAAGAGCCTACGACCATACCATGCTGAATATACCGGTTCTCGTCCGATCAC--------
5S107(prom): -----------------------------ATAGCTTAACTTTCATCAAGCAAGAGCCTACGACCATACCATGCTGAATATACCGGTTCTCGTCCGATCAC--------
5S108(prom): ----------------------------AATAGCTTAACTTTCATCAAGCAAGAGCCTACGACCATACCATGCTGAATATACCGGTTCTCGTCCGATCAC--------

PolyA101(prom): -----------------------GTGATGAAAAAAAAACTGAAAAAGAGAAAAATAAGAAAATCTTCTAGAACGTTCCGAAACAGGAC--------------------
PolyA102(prom): ----------------------CGTGATGAAAAAAAAACTGAAAAAGAGAAAAATAAGAAAATCTTCTAGAACGTTCCGAAACAGGAC--------------------
PolyA103(prom): ---------------------GCGTGATGAAAAAAAAACTGAAAAAGAGAAAAATAAGAAAATCTTCTAGAACGTTCCGAAACAGGAC--------------------
PolyA104(prom): --------------------TGCGTGATGAAAAAAAAACTGAAAAAGAGAAAAATAAGAAAATCTTCTAGAACGTTCCGAAACAGGAC--------------------
PolyA105(prom): -------------------TTGCGTGATGAAAAAAAAACTGAAAAAGAGAAAAATAAGAAAATCTTCTAGAACGTTCCGAAACAGGAC--------------------
PolyA106(prom): ------------------TTTGCGTGATGAAAAAAAAACTGAAAAAGAGAAAAATAAGAAAATCTTCTAGAACGTTCCGAAACAGGAC--------------------
PolyA107(prom): -----------------CTTTGCGTGATGAAAAAAAAACTGAAAAAGAGAAAAATAAGAAAATCTTCTAGAACGTTCCGAAACAGGAC--------------------
PolyA108(prom): ----------------CCTTTGCGTGATGAAAAAAAAACTGAAAAAGAGAAAAATAAGAAAATCTTCTAGAACGTTCCGAAACAGGAC--------------------

CG101(prom): -------------------------CAGCTGGAGCTCTCCTGTATGAGGGATCTGTTGATTCCAGCTGGGAGGTGTCTGCTACTCAGGAG------------------
CG102(prom): ------------------------CCAGCTGGAGCTCTCCTGTATGAGGGATCTGTTGATTCCAGCTGGGAGGTGTCTGCTACTCAGGAG------------------
CG103(prom): -----------------------GCCAGCTGGAGCTCTCCTGTATGAGGGATCTGTTGATTCCAGCTGGGAGGTGTCTGCTACTCAGGAG------------------
CG104(prom): ----------------------TGCCAGCTGGAGCTCTCCTGTATGAGGGATCTGTTGATTCCAGCTGGGAGGTGTCTGCTACTCAGGAG------------------
CG105(prom): ---------------------ATGCCAGCTGGAGCTCTCCTGTATGAGGGATCTGTTGATTCCAGCTGGGAGGTGTCTGCTACTCAGGAG------------------
CG106(prom): --------------------GATGCCAGCTGGAGCTCTCCTGTATGAGGGATCTGTTGATTCCAGCTGGGAGGTGTCTGCTACTCAGGAG------------------
CG107(prom): -------------------AGATGCCAGCTGGAGCTCTCCTGTATGAGGGATCTGTTGATTCCAGCTGGGAGGTGTCTGCTACTCAGGAG------------------
CG108(prom): ------------------CAGATGCCAGCTGGAGCTCTCCTGTATGAGGGATCTGTTGATTCCAGCTGGGAGGTGTCTGCTACTCAGGAG------------------
```

Figure S2: “With-promoter” looping sequences used in this work. Colors are the same as in Fig. S1. Note that these sequences are shorter versions of those in Fig. S1, so dashes indicate missing bases relative to the 108-bp version of each sequence given in Fig. S1. The $O_{id}$ operator is immediately $5'$ to these sequences, and has the sequence $5'$-AATTGTGAGCGCTCACAATT-$3'$; the *lac*UV5 promoter, $5'$-TTTACAATTAATGCTTCCGGCTCGTATAATGTGTGG-$3'$, is immediately $3'$ to these sequences, followed immediately by the $O_2$ operator, $5'$-GGTTGTTACTCGCTCACATTT-$3'$.

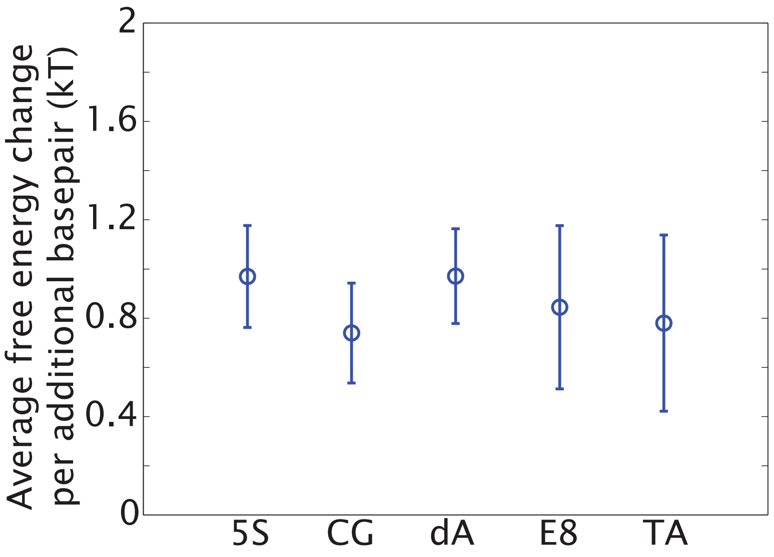


Figure S3: Sequence-dependent twist stiffness. In our data, differences in torsional stiffness between sequences would manifest as different amplitudes in the loop-length-dependent oscillations—that is, larger differences between peaks and troughs, or equivalently, different steepnesses of the slopes of the oscillations between peaks and troughs. One possible method for asking if we observe any such sequence-dependent changes in torsional stiffness is to fit the data of J-factors versus loop length in Fig. 2 in the main text to the functional form that has been derived for cyclization J-factors as a function of length (see Ref. [5]), even though the boundary conditions of looping and cyclization are very different, and discuss an "apparent" DNA stiffness. However, due to the lack of sufficient data in the troughs of the oscillations for all but the E8 and TA sequences, the errors on such a fitting attempt were too large for us to comment on the apparent DNA stiffness using this method. A second possible method for investigating a potential sequence-dependent twist stiffness is to calculate the average change in looping free energy (related to the looping J-factor through Eq. 1 in the main text) between loops of $n$ basepairs and loops of $n-1$ basepairs, for each sequence. This gives a measure of the amplitude of the length-dependent oscillations for each sequence. If the five sequences we examine differ in twist stiffness, we would expect the average change in looping free energy per basepair added to the loop to be different for the different sequences, in that stiffer sequences would have larger oscillations (or steeper slopes). We show such a calculation here, that is, the average change in free energy per basepair added to the loop. The five sequences show the same change in free energy as a function of additional basepairs, suggesting that they may share the same torsional stiffnesses, though again with limited data it is difficult to make conclusive statements. Data shown here are for no-promoter loops only, and the E8 and TA data are for 101-108 bp loop lengths only, for consistency with the other sequences.

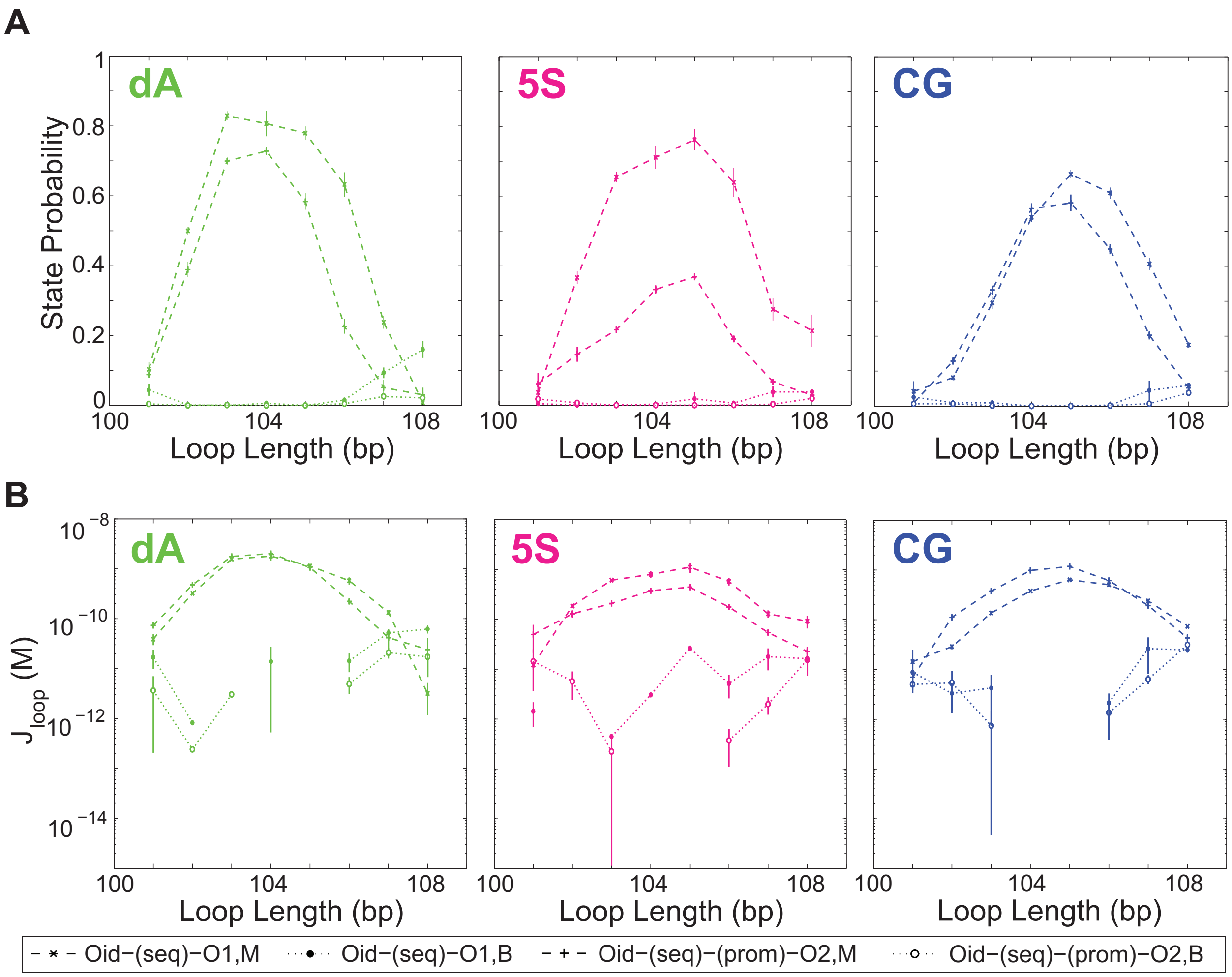


Figure S4: Looping probabilities **(A)** and J-factors **(B)** for the "middle" (M) and "bottom" (B) states separately. See Fig. 3 and the Supplementary Material of [1] for corresponding data for the E8 and TA sequences. Note that because of the different operators in the no-promoter versus with-promoter data, in some cases the no-promoter data has a lower looping probability than the with-promoter data, but yet a larger J-factor.

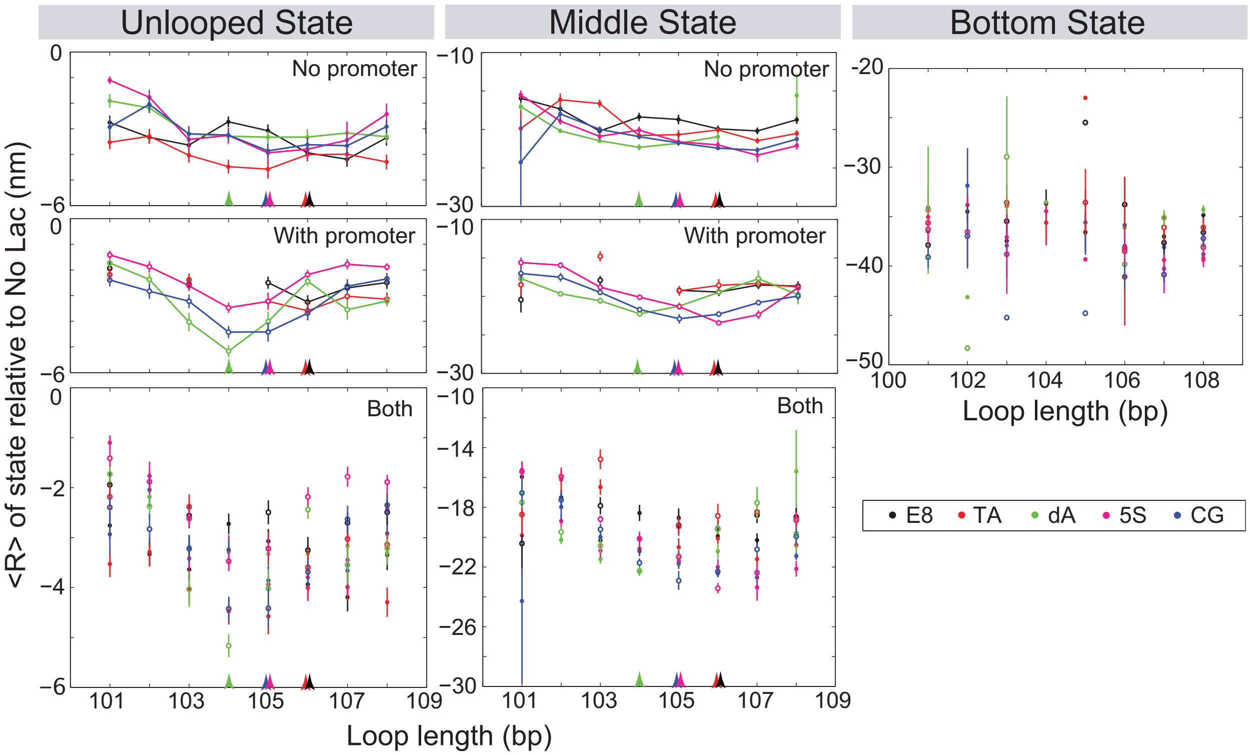


Figure S5: Tether lengths of looped and unlooped states as a function of loop length and sequence. The y-axes here and in the next figure are population averages of the difference in RMS between each bead's tether in the absence of protein, and the indicated state in the presence of protein (as described in the Supplementary Material of [1], there is sufficient tether-to-tether variability in the absence of protein, which we attribute to the variability in the diameters of the beads we use, that trends in tether lengths in the presence of protein are only observable when normalized to the length of each tether in the absence of protein). The bottom state has very poor statistics for most sequences and so should be considered indicative only of the ballparks of tether lengths we observe for that state. For the unlooped and middle states, however, we observe a modulation of tether length with loop length, with the shortest tether lengths for both states occurring near the maximum of looping (indicated for each sequence by the colored arrows at the bottoms of the plots). As argued more extensively in [1], we believe the reduction in tether length in the unlooped state in the presence of protein, compared to the tether length in the absence of protein, is due to the bending of the operators induced by the Lac repressor protein. The Lac repressor is known to bend the DNA of the $O_{id}$ operator by 45 degrees [6]; our previous work suggests that a bound Lac repressor also bends the other operators but to a lesser degree, with the extent of bending directly proportional to the strength of the operator. At the repressor concentration we use here, the unlooped state should be primarily composed of the doubly-bound state [1], meaning that the two operators are both bent by bound repressor. When these bends are in-phase, the tether length is shortest (and also the looping probability is highest, because the operators are in-phase). We believe this explains the modulation of tether length in the unlooped state. A similar argument can be made for the modulation of the middle looped state, regarding the relative phases of the tangents of the DNA exiting the loop.

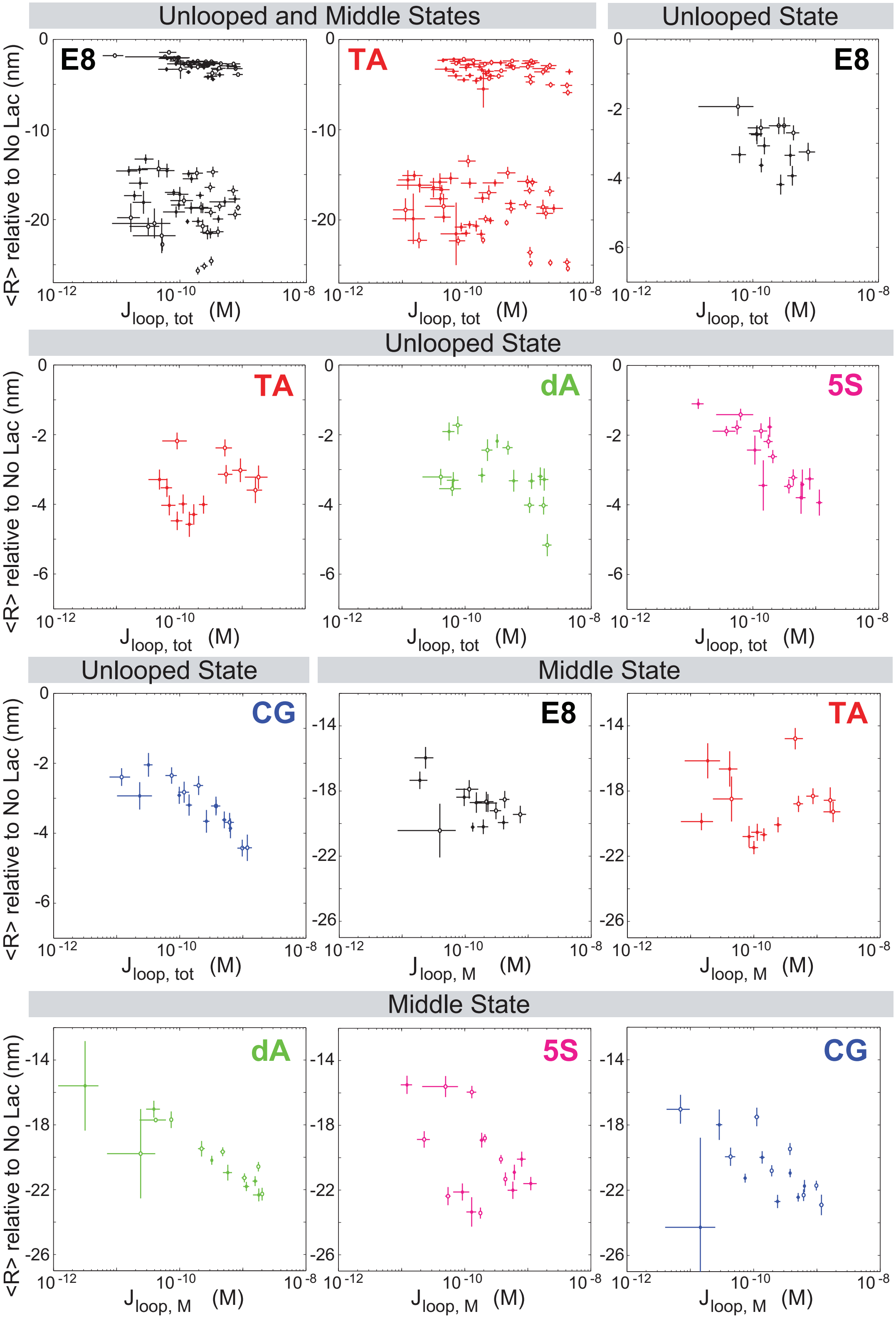


Figure S6: Tether length as a function of looping J-factor. Here as in the previous figure we show tether lengths for the indicated state normalized by each bead's RMS in the absence of protein, but as a function of J-factor, rather than loop length. Unlooped state tether lengths are always plotted versus the total J-factor, whereas middle state tether lengths

are always plotted versus the J-factor for the middle state. For completeness the first two panels show all data for both unlooped and middle states for E8 and TA; in the rest of the figure, only loop lengths 101-108 bp are shown for these two sequences, for consistency with the other sequences. As can also be seen in the previous figure, in general the length of the tether in both the unlooped and middle looped states is shorter at larger J-factors for a particular sequence. However, this trend is sharper for some sequences more than others: for example, the poly(dA:dT)-rich sequence exhibits a very tight correlation between the tether length of the middle state and the J-factor of the middle state, whereas 5S and CG exhibit a higher correlation in the unlooped state than the middle state. It is interesting to consider how the sequence of the loop might influence the length of the tether in the unlooped state, when no loop has formed (see, for example, CG with promoter versus 5S with promoter, where the latter is consistently longer than the former—perhaps seen most clearly in the previous figure). We do not see a sequence dependence to the tether lengths in the absence of repressor, ruling out the possibility of a detectable intrinsic curvature to the CG sequence. We speculate instead that CG alters the trajectory of the DNA as it exits the bend in the operators in the unlooped state, compared to the trajectory when the sequence next to the operators is 5S, leading to a consistent difference in unlooped tether length. Finally we note that there is no detectable systematic effect of the promoter on tether length: the TA data cluster by with- versus no-promoter, but only along the x-axis, because the presence of the promoter significantly increases the J-factor of the TA sequence. It does not significantly change the range of tether lengths exhibited by that sequence.

---

# References


[1] Johnson S, Lindén M, Phillips R (2012) Sequence dependence of transcription factor-mediated DNA looping. Nucleic Acids Res 40: 7728-7738.

[2] Simpson RT, Stafford DW (1983) Structural features of a phased nucleosome core particle. Proc Natl Acad Sci USA 80: 51-55.

[3] Field Y, Kaplan N, Fondufe-Mittendorf Y, Moore IK, Sharon E, et al. (2008) Distinct modes of regulation by chromatin encoded through nucleosome positioning signals. PLOS Comput Biol 4: e1000216.

[4] Yuan GC, Liu YJ, Dion MF, Slack MD, Wu LF, et al. (2005) Genome-scale identification of nucleosome positions in *S. cerevisiae*. Science 309: 627-630.

[5] Peters JP, Maher LJ (2010) DNA curvature and flexibility *in vitro* and *in vivo*. Quart Rev Biophys 43: 22-63.

[6] Lewis M, Chang G, Horton NC, Kercher MA, Pace HC, et al. (1996) Crystal structure of the lactose operon repressor and its complexes with DNA and inducer. Science 271: 1247–1254.
